# Long non-coding RNA HOTAIR knockdown enhances radiosensitivity through regulating microRNA-93/ATG12 axis in colorectal cancer

**DOI:** 10.1038/s41419-020-2268-8

**Published:** 2020-03-06

**Authors:** Yingqiang Liu, Xijuan Chen, Xiling Chen, Junqi Liu, Hao Gu, Ruitai Fan, Hong Ge

**Affiliations:** 1 Department of General Surgery, The Affiliated Tumor Hospital of Zhengzhou University, Zhengzhou, Henan China; 2 Department of Radiation Oncology, The Affiliated Tumor Hospital of Zhengzhou University, Zhengzhou, Henan China; 3 Department of Geriatric Medicine, The Second Affiliated Hospital of Zhengzhou University, Zhengzhou, Henan China; 4 Department of Radiation Oncology, The First Affiliated Hospital of Zhengzhou University, Zhengzhou, Henan China

**Keywords:** Cancer therapy, Cancer prevention

## Abstract

Colorectal cancer (CRC) is a global healthcare problem. Radioresistance is a huge setback for CRC radiotherapy. In this text, the roles and molecular mechanisms of long non-coding RNA HOTAIR in CRC tumorigenesis and radioresistance were further investigated. ATG12 mRNA, HOTAIR, and microRNA-93 (miR-93) levels were measured by quantitative reverse transcription polymerase chain reaction (RT-qPCR) assay. Protein levels of LC3 I, LC3 II, p62, ATG12, cleaved caspase 3, Bax, and Bcl-2 were detected by western blotting assay in cells and were examined by immunohistochemistry (IHC) assay in tissues. Cell survival fractions, viability, and apoptotic rates were determined by clonogenic survival assay, CCK-8 assay, and flow cytometry analysis, respectively. The relationships of HOTAIR, miR-93, and ATG12 were tested by bioinformatics analysis and luciferase reporter assay. Mouse xenograft tumor models were established to investigate the influence of HOTAIR knockdown on CRC radioresistance in vivo. We found that HOTAIR expression was markedly upregulated in plasma from CRC patients after radiotherapy and CRC cells after irradiation. HOTAIR knockdown, miR-93 overexpression, or ATG12 silencing weakened cell viability, induced cell apoptosis, inhibited cell autophagy, and enhanced cell radiosensitivity in CRC. HOTAIR exerted its functions by downregulating miR-93. Moreover, HOTAIR functioned as a molecular sponge of miR-93 to regulate ATG12 expression. ATG12 protein expression was markedly upregulated and associated with miR-93 and HOTAIR expression in CRC tissues. Furthermore, HOTAIR knockdown enhanced radiosensitivity of CRC xenograft tumors by regulating miR-93/ATG12 axis. In conclusion, HOTAIR knockdown potentiated radiosensitivity through regulating miR-93/ATG12 axis in CRC, further elucidating the roles and molecular basis of HOTAIR in CRC radioresistance.

## Introduction

Colorectal cancer (CRC) is a serious healthcare problem in the world, accounting for ~10% of all cancer cases and deaths^1^. It was estimated that more than 1.8 million new cases and 881,000 deaths from CRC occurred in 2018 globally, with a higher incidence rate in Europe^1^. The 5-year relative survival rate ranges from higher than 90% in CRC patients with early disease to about 10% in patients with advanced disease^2^. Radiotherapy is the cornerstone for the treatment of CRC, along with surgery and chemotherapy^3^. However, the existence and development of radioresistance is a great obstacle in the treatment of CRC^4,5^. Over the past decades, accumulating non-coding RNAs including long non-coding RNAs (lncRNAs) and microRNAs (miRNAs) have been found to be key regulators or potential biomarkers in tumor initiation, progression, and radioresistance of CRC^5–7^.

LncRNAs longer than 200 nucleotides (nt) in length and miRNAs with the size of about 21 nt are two major families of transcripts that lack protein-coding potential^8^. LncRNAs and miRNAs have been extensively studied for their regulatory roles in multiple cancer-related biological processes such as proliferation, apoptosis, and autophagy^9–11^. Moreover, some evidences disclose that lncRNAs can function as competing endogenous RNAs (ceRNAs) of miRNAs, resulting in the reduction of miRNA levels and increase of miRNA target levels^12,13^.

Homeobox transcript antisense intergenic RNA (HOTAIR), a well-studied lncRNA, has been widely reported as an oncogenic molecule in various cancers such as breast cancer, renal cancer, and nasopharyngeal cancer^14,15^. Previous studies showed that the depletion of HOTAIR could potentiate the radiosensitivity of some cancer cells such as breast cancer cells^16^ and cervical cancer cells^17^. Moreover, Yang et al.^18^ disclosed that HOTAIR knockdown suppressed cell proliferation, migration, and invasion, but promoted cell apoptosis and potentiated cell radiosensitivity in CRC. In this text, the roles and molecular mechanisms of HOTAIR in CRC tumorigenesis and radioresistance were further investigated.

Our present study demonstrated that HOTAIR knockdown reduced cell viability, promoted cell apoptosis, and inhibited cell autophagy by upregulating microRNA-93 (miR-93) and downregulating autophagy-related 12 (ATG12) in CRC. Moreover, HOTAIR loss enhanced radiosensitivity through regulating miR-93/ATG12 axis in CRC cells and CRC xenograft tumor models.

## Materials and methods

### Clinical samples and cell culture

Seventy-one patients diagnosed with CRC were recruited from the Affiliated Tumor Hospital of Zhengzhou University between 2012 and 2017. CRC tissues and adjacent normal tissues were collected from these patients through surgery. Partial tissue samples were snap frozen in liquid nitrogen and then stored at −80 °C till RNA extraction. Some specimens were fixed with formalin and embedded with paraffin for immunohistochemistry (IHC) and in situ hybridization (ISH) analysis. Blood samples were collected from 12 patients before or after radiotherapy. Then, plasma was isolated from blood through 10 min of centrifugation at 3000 r.p.m. Our study got approval of the Committees for the Ethical Review of Research at the Affiliated Tumor Hospital of Zhengzhou University and written informed consents from all patients.

Human normal colon epithelial cell line (FHC) and CRC cell lines (HT29, SW20, HCT116, and SW480) were purchased from American Type Culture Collection (Manassas, VA, USA). FHC cells were cultured in Dulbecco’s modified Eagle’s medium/F12 Medium (Thermo Scientific, Rockford, IL, USA) supplemented with 10 mM HEPES (Sigma-Aldrich, St. Louis, MO, USA), 10 ng/ml cholera toxin (Sigma-Aldrich), 0.005 mg/ml insulin (Sigma-Aldrich), 0.005 mg/ml transferrin (Sigma-Aldrich), 100 ng/ml hydrocortisone (Sigma-Aldrich), 20 ng/mL human recombinant epidermal growth factor (Sigma-Aldrich), and 10% fetal bovine serum (FBS, Thermo Scientific). HT29 and HCT116 cells were cultured in McCoy’s 5 A (Modified) Medium (Thermo Scientific) supplemented with 10% FBS (Thermo Scientific). SW620 and SW480 cells were grown in Leibovitz’s L-15 Medium (Thermo Scientific) containing 10% FBS (Thermo Scientific). FHC, HT29, and HCT116 were maintained in a humidified incubator containing 95% air and 5% CO_2_ at 37 °C. SW620 and SW480 cells were maintained in an incubator containing 100% air at 37 °C.

### Reagents and cell transfection

Small interfering RNAs (siRNAs) specifically targeting HOTAIR (siHOTAIR#1, siHOTAIR#2, and siHOTAIR#3) and a scramble control (scrambled), siRNA specific for ATG12 (siATG12) and its negative control (siNC), were purchased from GenePharma Co., Ltd (Shanghai, China). MiR-93 mimic (miR-93) and its negative control miR-NC and miR-93 inhibitor (anti-miR-93) and its negative control anti-miR-NC were ordered from Thermo Scientific, lnc. HOTAIR overexpression plasmid was constructed by Sangon Biotech Co., Ltd (Shanghai, China). All these oligonucleotides or plasmids were transfected into CRC cells using Lipofectamine 3000 reagent (Thermo Scientific), referring to the instructions of the manufacturer.

### RNA extraction and real-time quantitative PCR

Total RNAs were extracted from tissues and cells using mirVana miRNA isolation kit (Thermo Scientific) according to the instruction manual. Then, TaqMan miRNA assay (Thermo Scientific) was used to measure miR-93 expression level following the manufacturer’s instructions. U6 small nuclear RNA functioned as the internal control to normalize miR-93 expression. For the measurement of ATG12 mRNA level and HOTAIR level, M-MLV Reverse Transcriptase (Thermo Scientific) was used to conduct the reverse-transcription experiments and SYBR™ Green Master Mix (Thermo Scientific) was employed to perform the quantitative PCR analysis. Glyceraldehyde 3-phosphate dehydrogenase (*GAPDH*) served as the housekeeping gene to normalize the expression of ATG12 mRNA and HOTAIR. The quantitative primer sequences were listed as follows: 5′-ATTGCTGCTGGAGGGGAAGG-3′ (forward) and 5′-GGTTCGTGTTCGCTCTACTGC-3′ (reverse) for ATG12, 5′-CAGTGGGGAACTCTGACTCG-3′ (forward) and 5′-GTGCCTGGTGCTCTCTTACC-3′ (reverse) for HOTAIR, and 5′-CGGAGTCAACGGATTTGGTCGTAT-3′ (forward) and 5′-AGCCTTCTCCATGGTGGTGAGAC-3′ (reverse) for GAPDH.

### Western blot assay

Cells were lysed using ice-cold RIPA lysis buffer (Beyotime, Shanghai, China) supplemented with protease inhibitor cocktail (Roche Diagnostics, Mannheim, Germany). Then, cells were centrifuged for 15 min at 4 °C at the speed of 12,000 r.p.m. and cell supernatants containing proteins were collected. Next, protein concentration was determined through Bio-Rad Protein Assay (Bio-Rad Laboratories, Hercules, CA, USA). Subsequently, 30 μg of protein samples in each lane were separated by SDS-polyacrylamide gel electrophoresis and then transferred onto nitrocellulose membranes (Millipore, Bedford, MA, USA). After the blockage of nonspecific sites using 5% nonfat milk, the membranes were incubated overnight at 4 °C with primary antibodies against ATG12 (Abcam, Cambridge, UK), LC3B (Abcam), Bcl-2 (Abcam), Bax (Abcam), cleaved caspase 3 (Cell Signaling Technology, Danvers, MA, USA), and GAPDH (Abcam). Next, the membranes were probed for 1 h at room temperature with secondary antibody labeled with horseradish peroxidase (HRP) (Abcam). Finally, protein signals were visualized using Clarity Western ECL Substrate (Bio-Rad Laboratories) and quantified using Quantity One software Version 4.1.1 (Bio-Rad Laboratories).

### Luciferase reporter assay

Partial sequences of HOTAIR and ATG12 3′-untranslated region (3′-UTR) containing predicted miR-93 target sites were subcloned into psiCHECK-2 vector by Hanbio Biotechnology Co., Ltd (Shanghai, China), to produce HOTAIR-Wt reporter and ATG12-Wt reporter, respectively. Also, HOTAIR-Mut and ATG12-Mut reporters containing mutant miR-93 binging sites were constructed by Hanbio Biotechnology Co., Ltd. Then, cells were co-transfected with corresponding reporter vector and miR-93 mimic or miR-NC. Forty-eight hours later, cells were collected and lysed for luciferase reporter assay using a dual luciferase reporter assay system (Promega, Madison, WI, USA).

### Clonogenic survival assay

Transfected CRC cells were plated into six-well plates in triplicate and incubated for 24 h at 37 °C. After mock irradiated (0 Gy) or irradiated with different doses (2, 4, 6, 8 Gy) of ionizing radiation (IR), cells were maintained for about 2 weeks at 37 °C, to allow for colony formation. Next, colonies were fixed with 4% paraformaldehyde for 15 min and stained with 0.1% crystal violet solution (Sigma-Aldrich) for 15 min. Finally, the number of positive colonies containing more than 50 cells was counted.

### Cell viability analysis

Cell viability was analyzed using a Cell Counting Kit-8 (CCK-8) assay kit (Dojindo Molecular Technologies, Rockville, MD, USA) following the protocols of the manufacturer. Briefly, transfected cells were seeded into 96-well plates in triplicate and incubated for 24 h prior to the treatment with sham or 4 Gy of IR. At 24 h after irradiation, 10 μl of CCK-8 solution was added into each well and incubated for 3 h. Finally, cell absorbance was determined at 450 nm.

### Cell apoptosis detection

Cell apoptotic rate was determined using an eBioscience™ Annexin V-FITC Apoptosis Detection Kit (Thermo Scientific) referring to the instruction of the manufacturer. Briefly, transfected cells were plated into six-well plates in triplicate and incubated for 24 h prior to the treatment with sham or 4 Gy of IR. At 24 h post irradiation, cells were collected and then stained using Annexin V-FITC and propidium iodide solution. Finally, cell apoptotic rates were measured using a flow cytometry (FACScan; BD Biosciences, San Jose, CA, USA).

### IHC assay

Protein expression patterns of ATG12, LC3 II, and cleaved caspase 3 in tissues were assessed by IHC assay. Briefly, tissue sections were sequentially treated following the procedures of deparaffinage, rehydration, antigen retrieval, endogenous peroxidase blockage, and nonspecific signal blocking. Then, tissue slides were probed overnight at 4 °C with primary antibodies against ATG12, LC3 II, and cleaved caspase 3, and incubated for 1 h at room temperature with HRP-labeled secondary antibody. Next, these sections were visualized using 3,3’-diaminobezidin (DAB) substrate, counterstained with hematoxylin, and imaged. ATG12 expression level was assessed by the scores of positively labeled cells containing brown particles, which was recorded as: 0 (positive rate < 25%), 1 (positive rate: 25–50%), 2 (positive rate: 51–75%), and 3 (positive rate > 75%).

### ISH assay

Expression and localization patterns of HOTAIR in tissues were measured through ISH assay kit (FOCOFISH, Guangzhou, China) and custom-made probe following the protocols of the manufacturer. Briefly, after routine ISH treatment, tissue sections were incubated with HRP-conjugated labeling molecule, visualized using diaminobenzidine tetrahydrochloride (DAB) substrate, counterstained with hematoxylin staining solution, and imaged.

### In vivo radiation sensitivity experiments

Animal experiments were conducted with the approval of Institutional Committee for Animal Research of the Affiliated Tumor Hospital of Zhengzhou University and performed following the guide for the care and use of laboratory animals recommended by National Institutes of Health. Lentiviruses containing HOTAIR knockdown fragment (shHOTAIR) and its negative control were customized from Hanbio Biotechnology Co., Ltd. CRC xenograft tumor models were established by subcutaneous injection of 5 × 10^6^ SW480 cells infected with control lentiviruses or shHOTAIR lentiviruses into the flanks of male athymic nude mice (6 weeks old, Laboratory Animal Center of Henan, Zhengzhou, China) with 8 mice in each group. Ten days later, mice were exposed to 10 Gy of IR once. Tumor volume was monitored using a caliper at day 7, 10, 13, 16, 19, 22, 25, and 28 after injection, and calculated using the formula of (length × width^2^)/2. Tumors were resected and weighed at day 28 after injection. HOTAIR and miR-93 levels in CRC xenograft tumors were determined by quantitative reverse transcription polymerase chain reaction (RT-qPCR) assay. Protein expression patterns of ATG12, LC3 II, and cleaved caspase 3 were estimated by IHC assay.

### Statistical analysis

All in vitro experiments were repeated more than three times and animal experiments were conducted once with eight mice in each group. Data were presented as mean ± SD. Difference analysis was conducted using one-way analysis of variance (for multiple data) or Student’s *t*-test (for two group data) with *P* < 0.05 as statistically significant. Survival analysis was conducted using Kaplan–Meier method and log-rank test.

## Results

### HOTAIR was highly expressed in CRC tissues and cells, and HOTAIR expression was markedly upregulated in plasma samples from CRC patients after radiotherapy and CRC cells after IR treatment

**Fig. 1:**
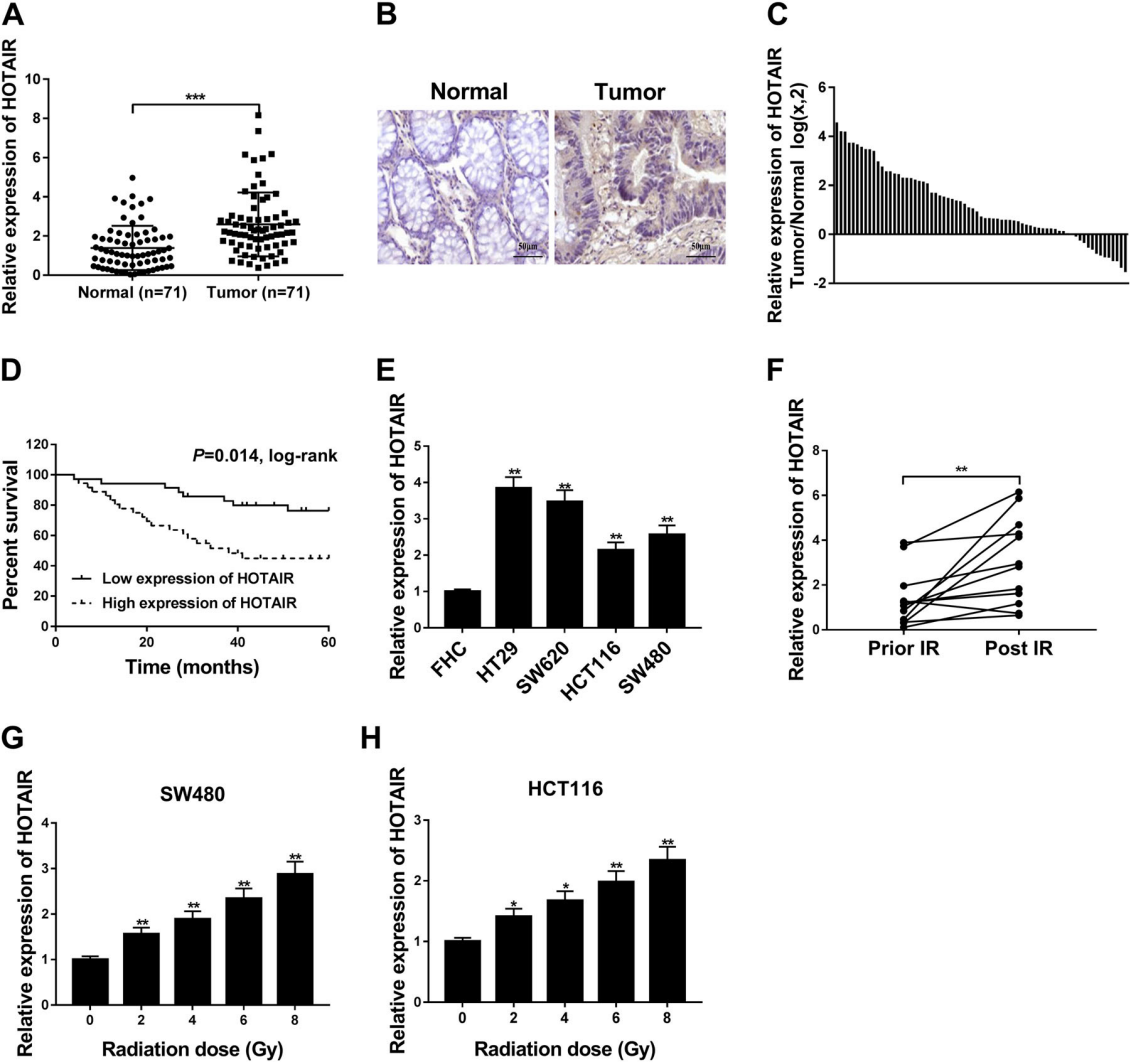
HOTAIR was highly expressed in CRC tissues and cells, and HOTAIR expression was markedly upregulated after IR treatment in CRC. **a**, **c** HOTAIR level was determined by RT-qPCR assay in 71 pairs of CRC tissues and corresponding normal tissues. **b** Representative images of ISH analysis for HOTAIR in CRC tissues and adjacent normal tissues. **d** Kaplan–Meier survival analysis of 71 CRC patients according to the difference of HOTAIR expression. **e** HOTAIR expression was measured by RT-qPCR assay in FHC, HT29, SW620, HCT116, and SW480 cells. **f** HOTAIR level was measured by RT-qPCR assay in plasma samples of 12 CRC patients before and after radiotherapy. **g**, **h** HOTAIR level was detected in SW480 and HCT116 cells treated with different doses (0, 2, 4, 6, or 8 Gy) of IR. *P < 0.05.

First, RT-qPCR assay and ISH analysis disclosed that HOTAIR was highly expressed in CRC tissues (*n* = 71) compared with adjacent normal tissues (*n* = 71) (Fig. 1a, b). ISH analysis also showed that HOTAIR was mainly distributed in the cytoplasm (Fig. 1b). Moreover, HOTAIR expression was remarkably increased in the majority (57/71) of CRC tissues than that in corresponding normal tissues (Fig. 1c). CRC clinical samples were divided into HOTAIR high-expression group (*n* = 36) and HOTAIR low-expression group (*n* = 35) with the median value of HOTAIR in CRC tissues as the cutoff point. Subsequent Kaplan–Meier survival analysis disclosed that CRC patients with high HOTAIR expression had a poor prognosis (Fig. 1d). Also, a notable increase of HOTAIR level was observed in CRC cells (HT29, SW620, HCT116, and SW480) relative to FHC cells (Fig. 1e). Next, the link of HOTAIR expression and radioresistance was further examined in CRC. As presented in Fig. 1f, HOTAIR level was markedly increased in most (11/12) of the plasma samples from CRC patients after radiotherapy, compared with those from corresponding patients before radiotherapy. Moreover, IR treatment at the dose of 2–8 Gy induced a dose-dependent elevation of HOTAIR level in SW480 and HCT116 cells (Fig. 1g, h).

### HOTAIR knockdown potentiated the radiosensitivity of CRC cells

**Fig. 2:**
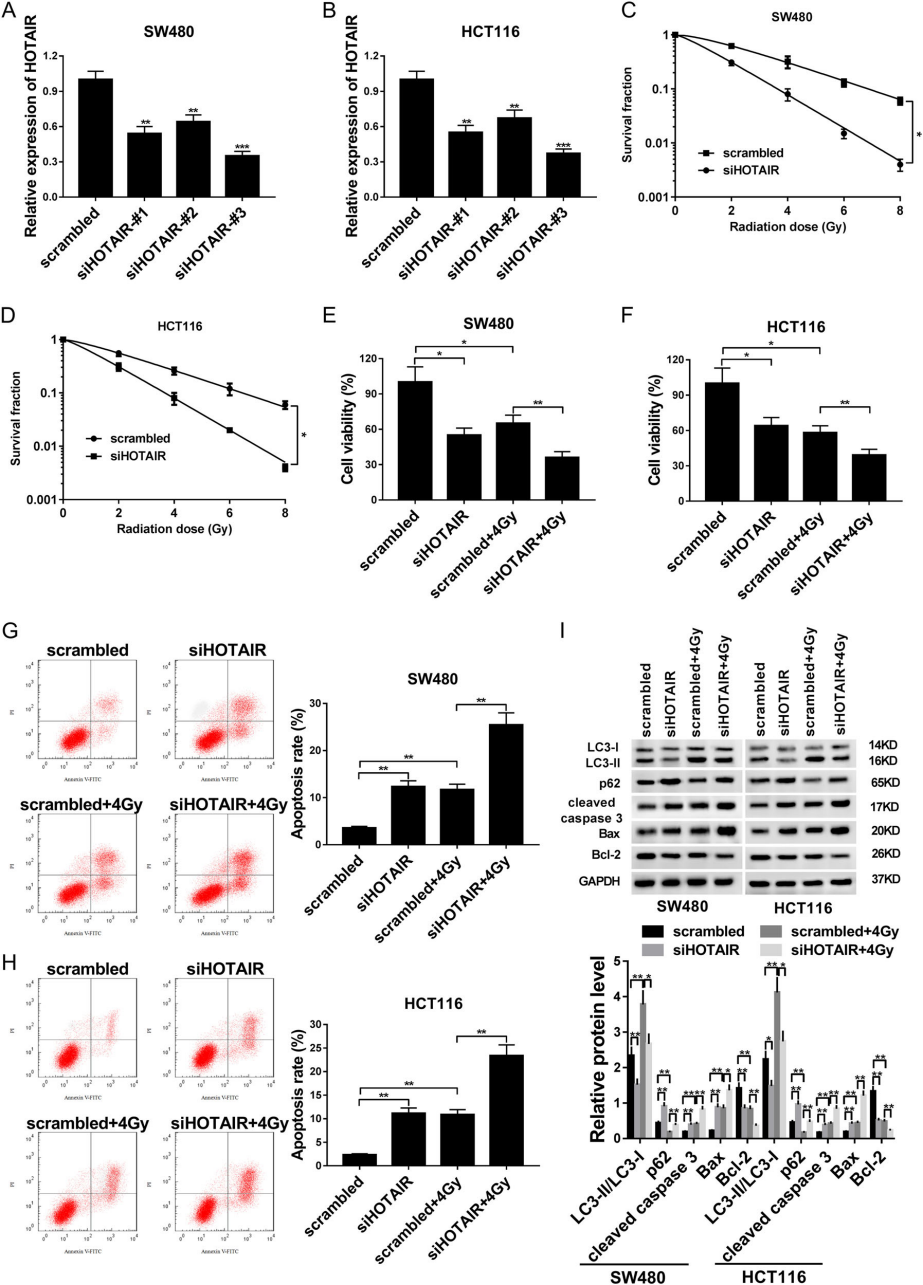
HOTAIR knockdown potentiated radiosensitivity of CRC cells. **a**, **b** sw480 and HCT116 cells were transfected with siHOTAIR#1, siHOTAIR#2, siHOTAIR#3, or a scramble control siRNA (scrambled). Forty-eight hours later, HOTAIR level was measured by RT-qPCR assay. **c**, **d** SW480 and HCT116 cells were transfected with siHOTAIR or its scramble control. At 24 h after transfection, cells were exposed to 0, 2, 4, 6, or 8 Gy of IR and then maintained for about 2 weeks. Next, the number of positive colonies were measured using a microscope and the survival fractions were calculated. **e**–**i** SW480 and HCT116 cells were transfected with siHOTAIR or its scramble control for 24 h prior to the treatment with or without sham or 4 Gy of IR. **e**, **f** After 24 h of irradiation, cell viability was measured by CCK-8 assay. **g**, **h** Cell apoptotic rate was estimated using a flow cytometry at 24 h post irradiation. **i** Protein levels of LC3 I, LC3 II, p62, cleaved caspase 3, Bax, and Bcl-2 were determined at 24 h after irradiation. *P < 0.05.

To further investigate the function of HOTAIR, three siRNAs targeting HOTAIR (siHOTAIR#1, siHOTAIR#2, or siHOTAIR#3) and a scramble control siRNA (scrambled) were synthesized. Following transfection efficiency analyses showed that the transfection of siHOTAIR#1, siHOTAIR#2, or siHOTAIR#3 could trigger the notable reduction of HOTAIR level in SW480 and HCT116 cells compared with that in scrambled group (Fig. 2a, b). Among these siRNAs, siHOTAIR#3 (siHOTAIR) was used for the following loss-of-function experiments due to its better knockdown efficiency. Subsequent radiation clonogenic survival assay showed that IR treatment (2–8 Gy) induced a dose-dependent reduction of cell survival fractions in SW480 and HCT116 cells transfected with siHOTAIR or control siRNA (Fig. 2c, d). HOTAIR knockdown led to the remarkable reduction of survival fractions of SW480 and HCT116 cells when cells were exposed to the same dose of IR (Fig. 2c, d). Moreover, HOTAIR depletion led to the reduction of cell viability (Fig. 2e, f), LC3 II/LC3 I ration (Fig. 2i) and Bcl-2 (anti-apoptosis protein) protein level (Fig. 2i), and increase of cell apoptotic rate (Fig. 2g, h) and protein levels of p62 (Fig. 2i), cleaved caspase 3 (Fig. 2i), and Bax (pro-apoptosis protein) (Fig. 2i) in SW480 and HCT116 cells, suggesting that HOTAIR knockdown impaired cell viability, induced cell apoptosis, and inhibited cell autophagy in CRC. Moreover, our data revealed that IR exposure weakened cell viability (Fig. 2e, f) and induced cell apoptosis (Fig. 2g, h) and autophagy (Fig. 2i) in SW480 and HCT116 cells transfected with siHOTAIR or control siRNA. In addition, HOTAIR knockdown triggered the reduction of cell survival fractions and cell viability, and the increase of cell apoptotic rate in IR (4 Gy)-treated SW480 and HCT116 cells, suggesting that HOTAIR loss enhanced radiosensitivity of CRC cells (Fig. 2c–i). Furthermore, HOTAIR depletion weakened IR-induced autophagy in SW480 and HCT116 cells (Fig. 2i). In addition, we demonstrated that LC3 II/LC3 I ratio was increased at first and then gradually reduced during 48 h after IR treatment with the highest level at 12 h after IR stimulation in SW480 cells transfected with siHOTAIR or scrambled siRNA (Supplementary Fig. 1). Protein level of cleaved caspase 3 was remarkably increased at 24 h after IR exposure in SW480 cells transfected with siHOTAIR or scrambled siRNA (Supplementary Fig. 1). These data suggested that mild autophagy could protect cell from detrimental conditions to a certain extent, whereas serious autophagy could induce cell apoptosis. In a word, these data suggested that HOTAIR knockdown might potentiate radiosensitivity of CRC cells by inhibiting autophagy.

### HOTAIR overexpression weakened the radiosensitivity of CRC cells by downregulating miR-93

**Fig. 3:**
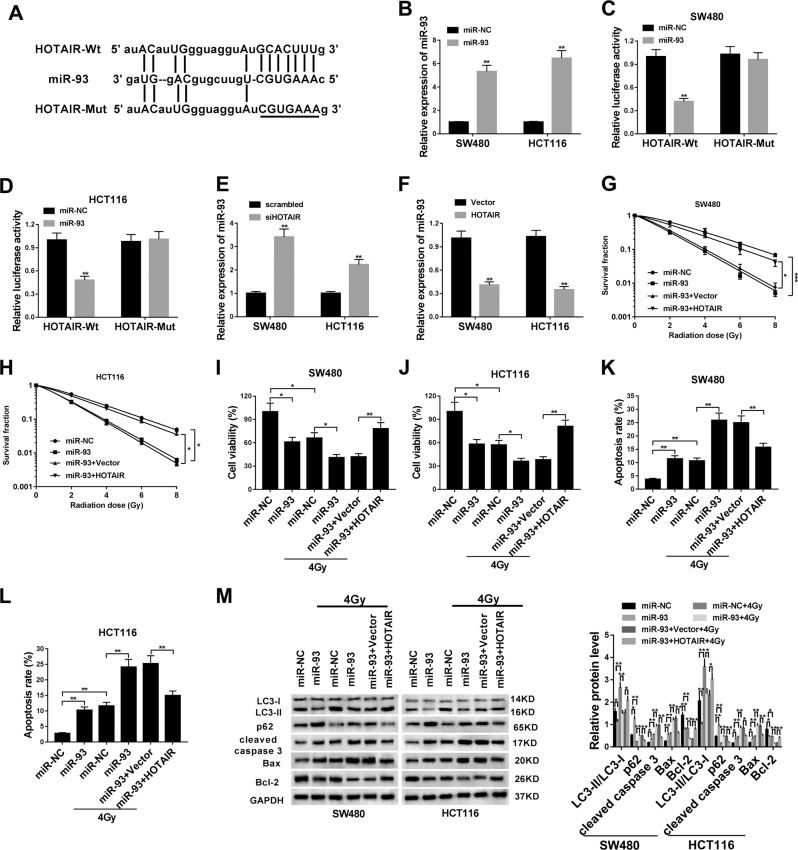
HOTAIR overexpression weakened the radiosensitivity of CRC cells by downregulating miR-93. **a** Predicted complementary sites between HOTAIR and miR-93, and mutant sites in HOTAIR-Mut reporter. **b** SW480 and HCT116 cells were transfected with miR-NC or miR-93 mimic. Forty-eight hours later, miR-93 level was measured by RT-qPCR assay. **c**, **d** SW480 and HCT116 cells were co-transfected with miR-NC or miR-93 mimic and HOTAIR-Wt or HOTAIR-Mut reporter, followed by the detection of luciferase activities at 48 h after transfection. **e**, **f** SW480 and HCT116 cells were transfected with siHOTAIR or its scrambled control, HOTAIR overexpression plasmid or its empty vector. Then, miR-93 level was measured by RT-qPCR assay at 48 h post transfection. **g**, **h** SW480 and HCT116 cells were transfected with miR-NC, miR-93 mimic, miR-93 + Vector, or miR-93 + HOTAIR for 24 h prior to the irradiation of various doses (0, 2, 4, 6, or 8 Gy) of IR. Then, cell survival fractions were measured by radiation clonogenic survival assay at 2 weeks after irradiation. **i**–**m** SW480 and HCT116 cells were transfected with miR-NC, miR-93 mimic, miR-93 + Vector, or miR-93 + HOTAIR for 24 h and then treated with or without 4 Gy of IR for 24 h, followed by the measurement of cell viability, cell apoptotic rate, protein levels of LC3 I, LC3 II, p62, cleaved caspase 3, Bax, and Bcl-2. *P < 0.05.

Next, bioinformatics analysis by StarBase online database showed that there existed some complementary sites between HOTAIR and “seed sequence” of miR-93 (Fig. 3a). To further validate this prediction, HOTAIR-Wt reporter containing putative miR-93-binding sites and HOTAIR-Mut reporter containing mutant miR-93-binding sites were constructed and then transfected into SW480 and HCT116 cells together with miR-NC or miR-93 mimic. Transfection efficiency analysis showed that the miR-93 level was markedly elevated in SW480 and HCT116 cells following the transfection of miR-93 mimic compared with miR-NC-transfected cells (Fig. 3b). Subsequent luciferase reporter assay revealed that miR-93 overexpression markedly reduced the luciferase activity of HOTAIR-Wt reporter in SW480 and HCT116 cells, but had no much influence on luciferase activity of HOTAIR-Mut reporter (Fig. 3c, d), suggesting that HOTAIR could bind with miR-93 through predicted binding sites. Next, RT-qPCR assay unveiled that HOTAIR knockdown induced the notable increase of miR-93 expression, whereas HOTAIR overexpression led to the remarkable downregulation of miR-93 level in SW480 and HCT116 cells (Fig. 3e, f). Functional analyses presented that miR-93 overexpression weakened cell viability (Fig. 3i, j), promoted cell apoptosis (Fig. 3k, l), and attenuated cell autophagic activity (Fig. 3m) in SW480 and HCT116 cells without IR treatment. Moreover, enforced expression of miR-93 resulted in the reduction of cell survival fractions and cell viability, and the increase of cell apoptosis in IR-treated SW480 and HCT116 cells, suggesting that miR-93 overexpression improved the radiosensitivity of CRC cells (Fig. 3g–m). In addition, our data disclosed that HOTAIR upregulation inhibited the increase of radiosensitivity caused by miR-93 in SW480 and HCT116 cells (Fig. 3g-m). Furthermore, miR-93 overexpression inhibited IR-induced autophagy in SW480 and HCT116 cells, whereas this effect of miR-93 was restored by upregulated HOTAIR (Fig. 3m). That was to say, HOTAIR overexpression weakened the radiosensitivity of CRC cells by downregulating miR-93 and relieving miR-93-mediated autophagy inhibition.

### HOTAIR positively regulated ATG12 expression through miR-93 in CRC cells

**Fig. 4:**
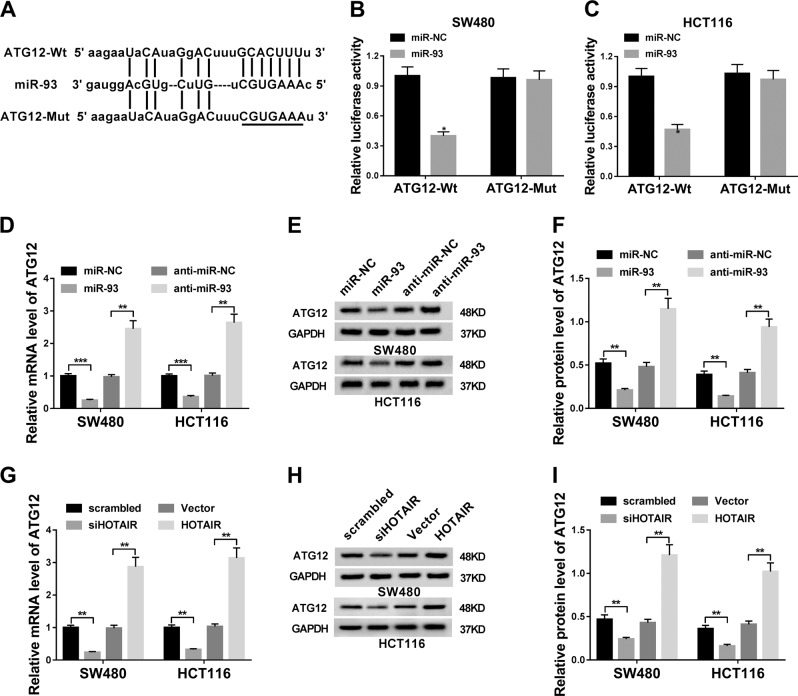
HOTAIR functioned as a ceRNA of miR-93 to regulate ATG12 expression. **a** MiR-93 target sites in ATG12 3′-UTR and mutant sites in ATG12-Mut reporter. **b**, **c** Luciferase activities were measured at 48 h post transfection in SW480 and HCT116 cells co-transfected with ATG12-Wt or ATG12-Mut reporter and miR-93 mimic or miR-NC. **d**–**f** SW480 and HCT116 cells were transfected with miR-NC, miR-93 mimic, anti-miR-NC, or anti-miR-93. Forty-eight hours later, ATG12 mRNA (**d**) and protein (**e**, **f**) levels were measured by RT-qPCR assay and western blot assay, respectively. **g**–**i** SW480 and HCT116 cells were transfected with siHOTAIR or its scramble control, HOTAIR overexpression plasmid or its empty vector. Forty-eight hours later, ATG12 mRNA (**d**) and protein (**e**) levels were measured by RT-qPCR assay and western blot assay, respectively. *P < 0.05.

Next, microRNA.org online prediction website was used to identify possible targets of miR-93. Among candidate targets, ATG12 was chosen considering its crucial roles in autophagy and radioresistance (Fig. 4a). To further demonstrate this prediction, ATG12-Wt reporter bearing miR-93-targeting sites and ATG12-Mut reporter containing mutant miR-93-binding sites were generated. Subsequent luciferase reporter assay showed that miR-93 overexpression mediated the notable downregulation of luciferase activity of ATG12-Wt reporter in SW480 and HCT116 cells, whereas the mutation of miR-93-binding sites in the 3′-UTR region of ATG12 reconstituted luciferase activity (Fig. 3b, c), verifying that miR-93 could bind with ATG12 3′-UTR by putative binding sites. Moreover, ATG12 mRNA and protein expression was strikingly reduced in SW480 and HCT116 cells following miR-93 overexpression (Fig. 4d–f). Conversely, miR-93 loss triggered the notable elevation of ATG12 expression at mRNA and protein levels in SW480 and HCT116 cells (Fig. 4d–f). In addition, ATG12 mRNA and protein expression was significantly reduced in HOTAIR-depleted SW480 and HCT116 cells, but was strikingly increased in HOTAIR-overexpressed cells (Fig. 4g–i). These data suggested that HOTAIR could act as a ceRNA of miR-93 to sequester miR-93 from ATG12, resulting in the reduction of miR-93 level and increase of ATG12 level in CRC cells.

### ATG12 protein expression was markedly upregulated and associated with miR-93 and HOTAIR expression in CRC tissues

**Fig. 5:**
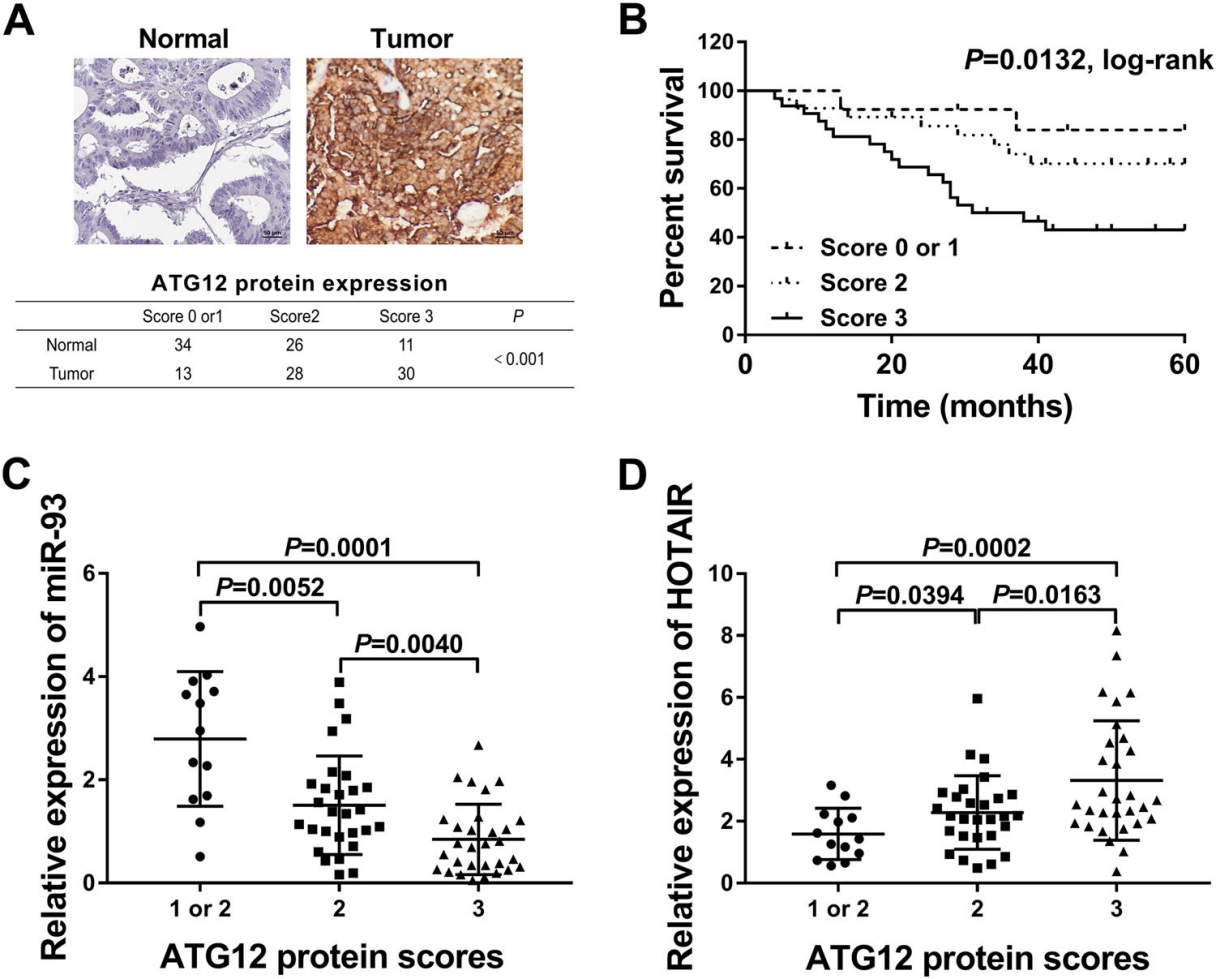
ATG12 protein expression was markedly upregulated and associated with miR-93 and HOTAIR expression in CRC tissues. **a** ATG12 protein expression pattern in CRC tissues and adjacent normal tissues were detected by IHC assay. **b** Kaplan–Meier survival analysis of CRC patients according to the difference of ATG12 protein scores. **c**, **d** MiR-93 or HOTAIR expression pattern in CRC tissues according to the difference of ATG12 protein scores. *P < 0.05.

Next, IHC analyses showed that ATG12 protein level was remarkably increased in CRC tissues compared with adjacent normal tissues (Fig. 5a). Kaplan–Meier survival analysis manifested that CRC patients with high ATG12 expression had a short survival (Fig. 5b). Moreover, ATG12 protein expression was negatively associated with miR-93 expression and positively associated with HOTAIR level in CRC tissues (Fig. 5c, d).

### ATG12 knockdown improved the radiosensitivity of CRC cells

**Fig. 6:**
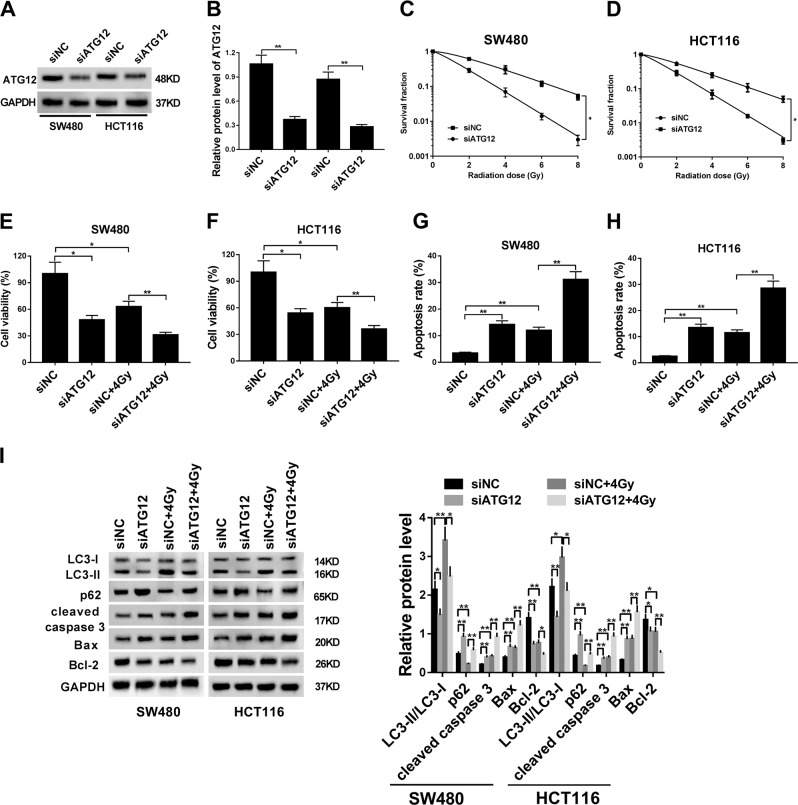
ATG12 knockdown potentiated the radiosensitivity of CRC cells by inhibiting autophagy. **a**, **b** Transfection efficiency analysis of siATG12 in SW480 and HCT116 cells through western blot assay at 48 h after transfection. **c**–**h** The effects of ATG12 knockdown on cell radiosensitivity, viability, and apoptosis were measured by radiation clonogenic survival assay (**c**, **d**), CCK-8 assay (**e**, **f**), and flow cytometry analysis (**g**, **h**). **i** The effects of ATG12 silencing alone or in combination with IR stimulation on protein expression of LC3 I, LC3 II, p62, cleaved caspase 3, Bax, and Bcl-2 were measured by western blot assay. *P < 0.05.

To further examine the function of ATG12 in CRC, siATG12 and its negative control siNC were synthesized. Following western blot assay demonstrated that the transfection of siATG12 triggered conspicuous reduction of ATG12 protein levels in SW480 and HCT116 cells (Fig. 6a, b), meaning that siATG12 could be used for the subsequent loss-of-function experiments. Radiation clonogenic survival assay indicated that ATG12 knockdown led to the notable reduction of cell survival fractions in SW480 and HCT116 cells treated with IR (Fig. 6c, d). Consistently, cell viability was reduced in IR-treated SW480 and HCT116 cells following the depletion of ATG12 (Fig. 6e, f). Moreover, ATG12 knockdown enhanced IR-induced apoptosis in SW480 and HCT116 cells (Fig. 6g–i). That was to say, ATG12 loss enhanced the radiosensitivity of CRC cells. Furthermore, ATG12 knockdown alleviated the aggressive phenotypes of CRC cells, as evidenced by the reduction of cell viability and increase of apoptotic rate and cleaved caspase 3 protein level in ATG12-depleted SW480 and HCT116 cells compared with siNC-transfected cells (Fig. 6e–i). Moreover, LC3 II/LC3 I ratio was reduced and p62 protein level was increased in SW480 and HCT116 cells following the knockdown of ATG12, indicating that ATG12 deficiency inhibited autophagy in CRC cells (Fig. 6i). Furthermore, ATG12 knockdown suppressed the elevation of cell autophagic activity induced by IR in SW480 and HCT116 cells (Fig. 6i). Taken together, these outcomes showed that ATG12 knockdown potentiated the radiosensitivity of CRC cells by inhibiting autophagy.

### HOTAIR knockdown enhanced radiosensitivity of CRC xenograft tumors

**Fig. 7:**
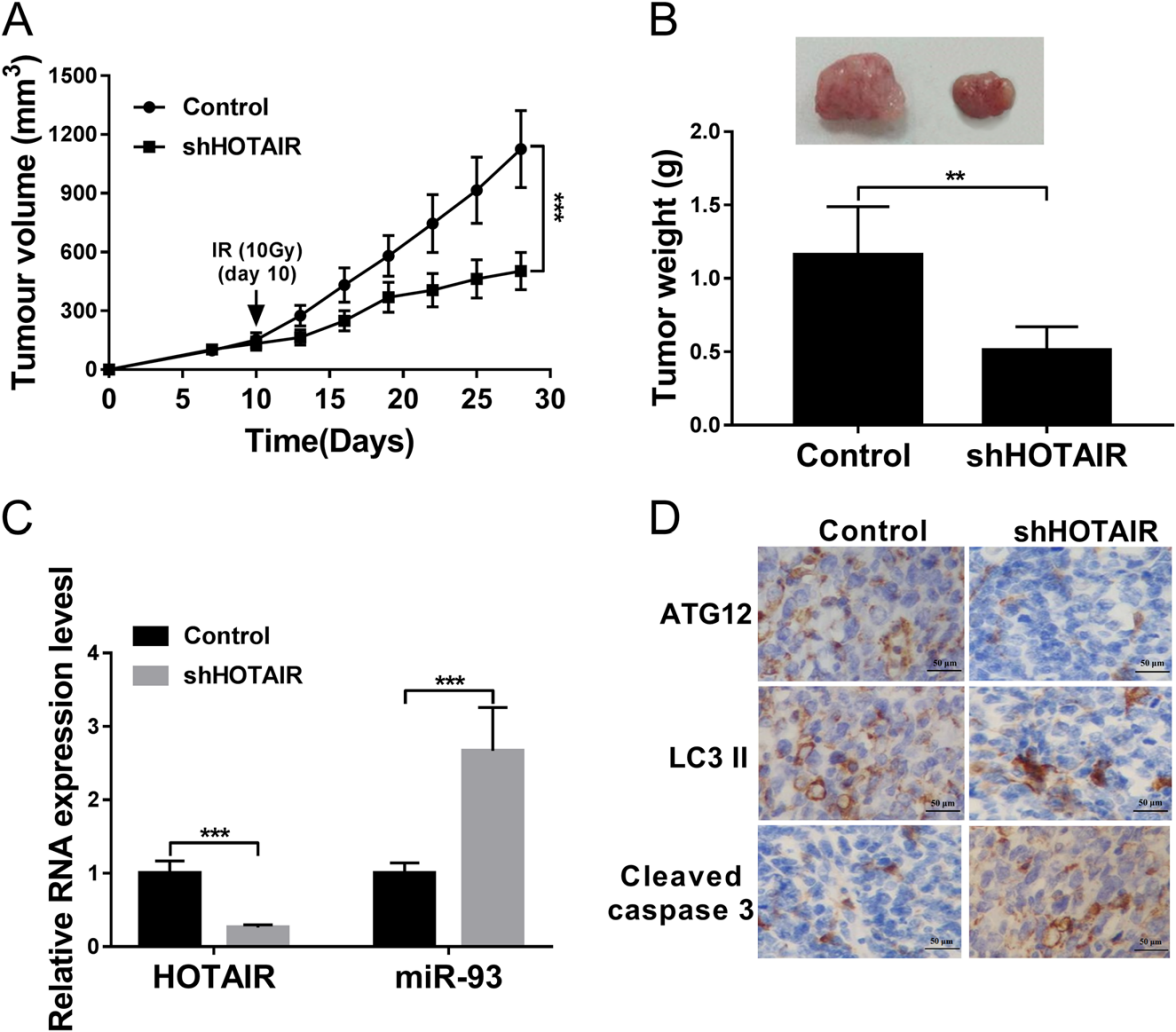
HOTAIR knockdown enhanced radiosensitivity of CRC xenograft tumors. **a**–**d** CRC xenograft tumor models were established by subcutaneous injection of SW480 cells infected with control lentiviruses or shHOTAIR lentiviruses into the flanks of mice. Ten days later, mice were irradiated with 10 Gy of IR once. **a** Tumor volume was monitored using a caliper at day 7, 10, 13, 16, 19, 22, 25, and 28 after injection. **b** Tumor weight was measured at day 28 after injection. **c** HOTAIR and miR-93 levels in CRC xenograft tumors were determined by RT-qPCR assay. **d** Protein expression patterns of ATG12, LC3 II, and cleaved caspase 3 were evaluated by IHC assay. *P < 0.05.

Next, we further demonstrated that CRC xenograft tumors with HOTAIR knockdown and IR treatment grew more slowly than IR-treated control tumors (Fig. 7a, b). Also, RT-qPCR assay demonstrated that the HOTAIR level was noticeably reduced and miR-93 level was obviously increased in IR-treated CRC xenograft tumors following the introduction of shHOTAIR lentiviruses (Fig. 7c). Moreover, IHC analyses showed that ATG12 and LC3 II protein expression was markedly reduced and cleaved caspase 3 protein level was strikingly increased in CRC xenograft tumors with HOTAIR knockdown and IR treatment than that in IR-treated control tumors (Fig. 7d). Based on these data, we might draw the conclusion that HOTAIR knockdown enhanced radiosensitivity by regulating miR-93/ATG12-mediated autophagy in CRC xenograft tumors.

## Discussion

CRC is a huge threat for human health and life with the third morbidity and the second mortality in all cancers worldwide^1^. Radioresistance is a major reason for the failure of radiotherapy in cancers. An in-depth understanding on radioresistance-related molecular mechanisms contributes to the improvement of radiotherapy outcomes.

In this text, we demonstrated that HOTAIR was highly expressed in CRC tissues and cells, which was in line with previous studies^18,19^. Also, consistent with prior reports^19,20^, our data disclosed that CRC patients with high HOTAIR expression had an unfavorable prognosis. In this text, we further demonstrated that HOTAIR expression was remarkably increased in plasma samples from CRC patients after radiotherapy and IR-stimulated CRC cells. Functional analysis revealed that HOTAIR knockdown resulted in the downregulation of cell viability and increase of cell apoptosis in CRC cells. Similar with our data, prior studies demonstrated that HOTAIR loss weakened the proliferative, migratory, and invasive capacities of CRC cells and induced CRC cell apoptosis^18,21,22^.

HOTAIR-mediated autophagy has been found to be an essential event in the development and progression of some cancers such as breast cancer^23^ and hepatocellular cancer^24^. Moreover, HOTAIR could affect chemoresistance by regulating autophagy in endometrial cancer^25^ and non-small cell lung cancer^26^. Hence, we further investigated whether HOTAIR could control radioresistance by regulating autophagy in CRC. Our present study demonstrated that HOTAIR depletion suppressed autophagy and weakened IR-induced autophagy in CRC cells. Moreover, HOTAIR depletion potentiated radiosensitivity of CRC cells in vitro, which was in accordance with a previous study^18^. In summary, these data disclosed that HOTAIR loss improved radiosensitivity of CRC cells by inhibiting autophagy. Similarly, a recent study pointed out that HOTAIR knockdown enhanced radiosensitivity by inhibiting autophagy in pancreatic cancer cells^27^. Also, we further demonstrated that HOTAIR loss enhanced IR-mediated anti-tumor effects in CRC xenograft tumors.

MiR-93, a member of miR-106b ~25 cluster^28^, has been found to be closely linked with tumor initiation, progression, and radioresistance in some malignancies^29–31^. Also, previous studies showed that miR-93 could function as a negative regulator of cell autophagy^32^. Moreover, bioinformatics analysis by StarBase online website showed that there were several complementary sites between HOTAIR and miR-93. In addition, microRNA.org online prediction analysis suggested that ATG12 was a potential target of miR-93. ATG12, a core component of autophagy-related complexes, has also been reported to be implicated in tumor initiation, progression, and radioresistance in some cancers such as cervical cancer^33^ and bladder cancer^34^. Moreover, miR-93 expression was markedly reduced in CRC tissues and cells, and was negatively associated with HOTAIR expression in CRC tissues (data not presented). In addition, miR-93 expression was notably downregulated in plasma samples of CRC patients after radiotherapy and CRC cells following IR treatment (data not presented).

Bao et al.^35^ demonstrated that HOTAIR loss-induced autophagy inhibition led to the reduction of cell viability and increase of cell apoptotic rate by regulating miR-454–3p/ATG12 or miR-454-3p/STAT3 axis in chondrosarcoma. Considering the interrelationships of HOTAIR, miR-93, and ATG12, we further investigated whether HOTAIR could exert its functions by regulating miR-93 and its downstream target ATG12 in CRC.

In this text, we demonstrated that HOTAIR inhibited miR-93 expression by direct interaction and ATG12 was a direct target of miR-93. Moreover, HOTAIR facilitated ATG12 expression in CRC cells, suggesting that HOTAIR functioned as a molecular sponge of miR-93 to induce ATG12 expression in CRC.

Functional analyses presented that miR-93 overexpression weakened cell viability, promoted cell apoptosis, and inhibited cell autophagy in CRC. Similarly, Tang et al.^36^ demonstrated that ectopic expression of miR-93 reduced proliferative, migratory, and invasive capacities of CRC cells and hampered CRC xenograft growth by targeting smad7. However, Yang et al.^37^ demonstrated that miR-93 overexpression curbed CRC cell proliferation and migration in vitro as well as impeded CRC xenograft growth in vivo, but had no much impact on CRC cell invasion and apoptosis. In addition, a recent report showed that miR-93 overexpression weakened CA3-AS1-mediated anti-proliferation, anti-invasion, and pro-apoptosis effects by inhibiting downstream target phosphatase and tensin homolog expression in CRC cells^38^.

Our present study further revealed that miR-93 overexpression potentiated radiosensitivity of CRC cells, as evidenced by the reduction of cell survival fraction and cell viability, and increase of cell apoptotic rate in IR-exposed cells following the upregulation of miR-93. Moreover, ectopic expression of miR-93 inhibited the increase of cell autophagic activity induced by IR in CRC cells. In addition, restoration experiments showed that HOTAIR overexpression abrogated the effects of miR-93 on cell survival, viability, apoptosis, and autophagy in IR-treated CRC cells, suggesting that HOTAIR overexpression weakened radiosensitivity of CRC cells by downregulating miR-93 and relieving miR-93-mediated autophagy inhibition.

Moreover, our study demonstrated that ATG12 protein expression was markedly upregulated in CRC tissues. Moreover, correlation analyses disclosed that ATG12 protein expression was negatively associated with miR-93 expression, but positively associated with HOTAIR expression in CRC tissues. In addition, ATG12 knockdown triggered the reduction of cell viability and cell autophagic activity, and increase of cell apoptotic rate in CRC. Moreover, ATG12 deficiency enhanced cell radiosensitivity and weakened IR-induced autophagy in CRC cells. In line with our results, a recent study showed that IR-induced autophagy inhibited cell apoptosis and reduced cell radiosensitivity in CRC, and miR-214 improved the radiosensitivity of CRC cells through inhibiting ATG12-mediated autophagy in vitro and in vivo^39^.

In addition, in vivo experiments demonstrated that HOTAIR knockdown potentiated IR-mediated anti-tumor effects by increasing miR-93 and cleaved caspase 3 expression, and reducing ATG12 and LC3 II expression in CRC xenograft tumors.

Taken together, our data demonstrated that HOTAIR depletion suppressed CRC tumorigenesis in vitro through regulating miR-93/ATG12-mediated autophagy. Moreover, HOTAIR knockdown potentiated the radiosensitivity of CRC cells in vitro and in vivo through upregulating miR-93 and downregulating ATG12-mediated autophagy. Our study provides a deep insight into the roles and molecular basis of HOTAIR in regulating CRC tumorigenesis and radioresistance, and suggests the potential values of HOTAIR in the prognosis, treatment, and radioresistance prevention of CRC. However, our conclusion needs to be further validated by inhibitors or activators of autophagy in vitro and in vivo.

## Supplementary information

Supplementary Figure 1

Supplementary Figure legend
