# Supplementary figures and images for: Long non-coding RNA HOTAIR knockdown enhances radiosensitivity through regulating microRNA-93/ATG12 axis in colorectal cancer
Source: Cell Death Dis. 2020 Mar 6;11(3):175. doi: 10.1038/s41419-020-2268-8 (PMC7060216; doi:10.1038/s41419-020-2268-8)

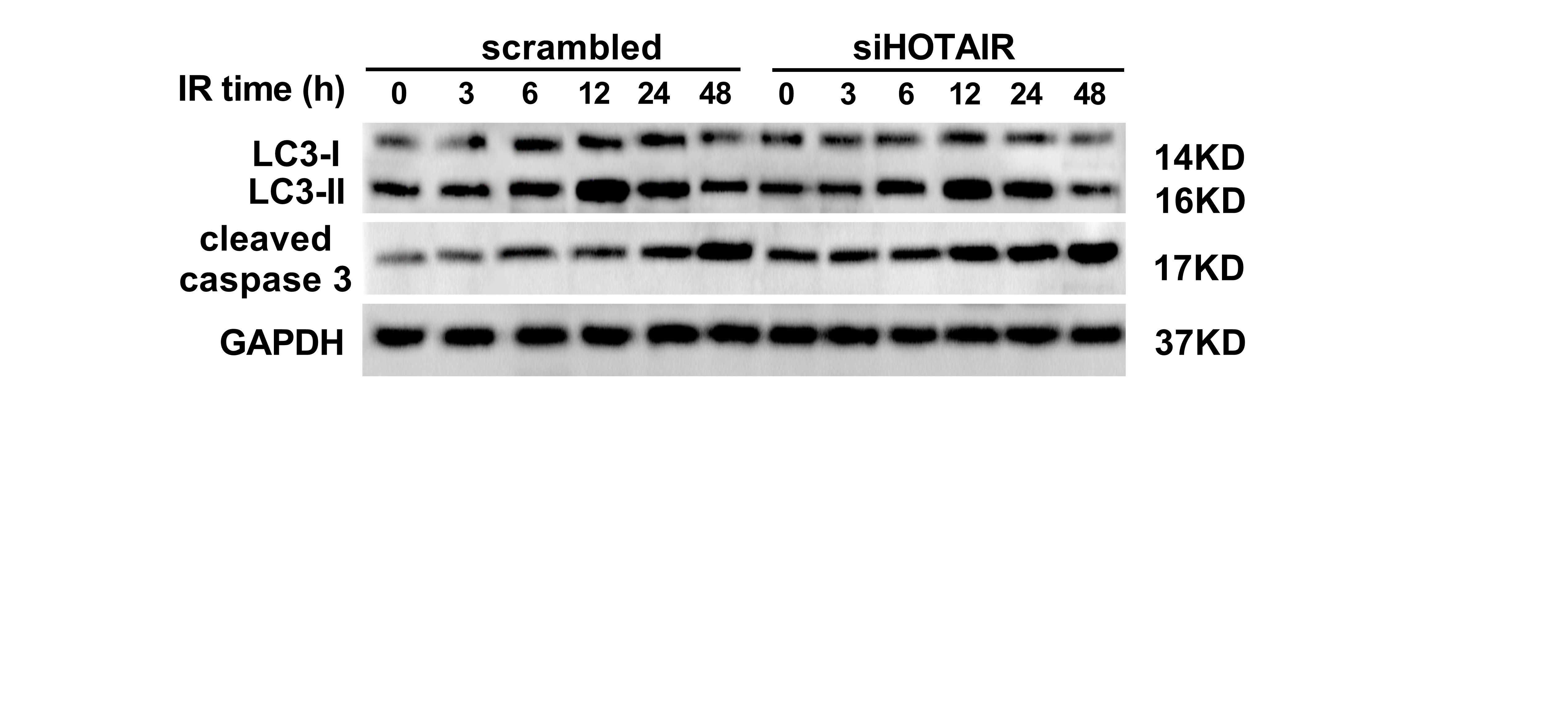

Supplement: Supplementary file 1 — Supplementary Figure 1 [file 41419_2020_2268_MOESM1_ESM.tif]
