# Supplementary material for: Long non-coding RNA HOTAIR knockdown enhances radiosensitivity through regulating microRNA-93/ATG12 axis in colorectal cancer
Source: Cell Death Dis. 2020 Mar 6;11(3):175. doi: 10.1038/s41419-020-2268-8 (PMC7060216; doi:10.1038/s41419-020-2268-8)
Supplement: Supplementary file 2 — Supplementary Figure legend [file 41419_2020_2268_MOESM2_ESM.docx]

**Supplementary Fig. 1 Protein levels of LC3 and cleaved caspase 3 in IR stimulated SW480 cells transfected with siHOTAIR or scrambled siRNA.**
